# Neck Circumference and Its Relation with Body Fat Percentage in Children 5–10 Years Old

**DOI:** 10.3390/children11070868

**Published:** 2024-07-17

**Authors:** Enrique Romero-Velarde, Karen G. Córdova-García, Laura C. Robles-Robles, Ingrid J. Ventura-Gómez, Clío Chávez-Palencia

**Affiliations:** 1 Instituto de Nutrición Humana, Centro Universitario de Ciencias de la Salud, Universidad de Guadalajara, Guadalajara 44340, Jalisco, Mexico; ingrid.ventura@alumno.buap.mx; 2 División de Pediatría, Hospital Civil de Guadalajara “Dr. Juan I. Menchaca”, Guadalajara 44340, Jalisco, Mexico; karen.cordova7214@alumno.udg.mx (K.G.C.-G.); cristina.robles@academicos.udg.mx (L.C.R.-R.); 3 División de Ciencias de la Salud, Centro Universitario Tonalá, Universidad de Guadalajara, Tonalá 45425, Jalisco, Mexico; clio.chavez@academicos.udg.mx

**Keywords:** neck circumference, body fat percentage, children, obesity

## Abstract

Background: Neck circumference (NC) has been proposed as an indicator of upper trunk adiposity and a potential indicator of metabolic risk. The objective was to evaluate NC and its correlation with body fat percentage (BF%) and other indicators of adiposity in children with normal weight, overweight, and obesity. Methods: In a cross-sectional study, 112 children 5 to 10 years of age were included in the outpatient clinic from a public hospital. Measures of weight and height to calculate BMI (kg/m^2^), NC, mid-upper arm circumference, waist circumference, and tricipital skinfold thickness. Body composition measurements were performed using an electrical bioimpedance device (BIA). The relationship between anthropometric variables and BF% obtained by BIA was determined using Spearman correlation tests. Multivariate models were constructed with BF% as the dependent variable and anthropometric parameters as independent. Results: In the entire group, there was a direct correlation between NC and BF% (r = 0.50, *p* < 0.001), but lost statistical significance in the case of normal weight. The relationship maintained its significance in subjects from the overweight and obesity groups. In multivariate models, BMI exhibited the highest correlation with BF%, followed by waist circumference and mid-upper arm circumference; for NC, the R^2^ value was 0.30 (*p* < 0.001). Conclusions: Neck circumference is useful in the screening of population groups with the advantage of not requiring any specialized instruments for its measurement other than a tape measure. BMI and waist circumference were the best indicators of general and central adiposity, respectively.

## 1. Introduction

Obesity is a public health problem worldwide; in Mexico, its prevalence among school-age children is 18.1% [1,2]. For several years, the most used tool in both clinical and population diagnoses has been body mass index (BMI), which is considered to be a good indicator of general adiposity [3]. However, BMI has limitations, including its inability to reflect the distribution of body fat [4]; therefore, complementary measurements, such as waist circumference (WC) and waist-to-hip ratio (WHR), which are associated with abdominal obesity and higher metabolic risk, have also been used to evaluate subjects with obesity [5].

Recently, neck circumference (NC) has been proposed as an indicator of upper trunk adiposity and as an alternative criterion for the diagnosis of overweight and obesity [6,7]. Different studies have reported a direct correlation between NC and BMI and other indicators of adiposity, such as WC, although the strength of the correlation varies among studies that include populations of children and adolescents [8,9,10,11,12]. Furthermore, some studies have indicated that the correlation is weaker and even non-significant when obese subjects are evaluated [8,13].

Due to its correlation with WC, NC has been considered to be a potential indicator of metabolic risk associated with the accumulation of visceral fat [14]. A recent systematic review reported that NC was consistently and significantly correlated with high-density lipoprotein cholesterol, insulin, and blood pressure. However, the authors recommended its use in conjunction with other metabolic risk indicators and not as a single indicator [15].

The correlation between NC and body fat percentage (%BF) evaluated using plethysmography and bioelectrical impedance (BIA) has also been reported, with r values between 0.44 and 0.67, although among females, these values reach up to 0.83 in the case of BIA at 11 years of age [9,10,16].

More frequently, correlations between NC and these anthropometric indicators have been reported for groups of children and adolescents in general and not according to body weight categories [11,17,18]. It is important to evaluate whether the correlation between NC and variables routinely used in the evaluation and diagnosis of overweight and obesity, such as BMI and WC, is consistent in children with normal weight, overweight, or obesity. As such, NC could be used as an alternative measurement to identify children and adolescents at metabolic risk, particularly because of its greater accessibility during anthropometric evaluation. Therefore, the objective of this study was to evaluate NC and its correlation with %BF and other indicators of adiposity in children with normal weight, overweight, and obesity in a sample population with a high prevalence of obesity, such as Mexico. The identification of adiposity indicators is crucial for early intervention and implementation of preventive measures.

## 2. Materials and Methods

This cross-sectional study included children 5 to 10 years of age, recruited from the outpatient clinic of the Pediatrics Division of the Hospital Civil de Guadalajara “Dr. Juan I. Menchaca” between October 2020 and May 2021. The outpatient clinic serves pediatric patients in general, as well as patients from different subspecialty clinics. Apparently healthy children without acute illnesses were recruited with parental consent. Individuals with systemic diseases, such as diabetes mellitus and hypothyroidism, genetic or congenital diseases, a history of chronic steroid use by any means, or those presenting with vomiting or diarrhea 5 days before the evaluation were excluded. A correlation coefficient between NC and BMI (0.6) was used to calculate the sample size, with a minimum of 75 subjects. Consecutive children were included and divided into 3 groups according to BMI: normal weight (−2 to 1 standard deviation[s] [SD]); overweight (>1 to 2 SDs); and obesity (>2 SDs) using WHO criteria and reference tables [19].

Two researchers visited the outpatient clinic during the week to identify potentially eligible subjects for inclusion in this study. Subjects were invited to participate and, for those who accepted, general data, including age, sex, family, and sociodemographic characteristics, were obtained, in addition to measuring weight and height to calculate BMI (kg/m^2^), NC, mid-upper arm circumference (MUAC), WC, and tricipital skinfold thickness (TSF).

Body weight, with excess clothing and shoes removed, was measured using a scale (model 700, Seca, Hamburg, Germany). Height was measured using a stadiometer (model 220, Seca), ensuring the position of the head in the Frankfort plane, with the subjects keeping their feet and knees together, and with the back of the trunk, buttocks, and heels abutted to the posterior plane of the stadiometer. To measure WC, MUAC, and NC, a 6 mm wide Rosscraft metal measuring tape was used. Mid-upper arm circumference was measured in the middle of the left arm. Waist circumference was measured as the minimum circumference between the costal margin and iliac crest. Neck circumference was measured with the patient standing upright and the head in the Frankfort horizontal plane, with the tape placed at the midpoint of neck height. Tricipital skinfold thickness was measured using a Harpenden skinfold caliper (Baty International, Burgess Hill, UK) at mid-height of the right arm.

Subsequently, body composition measurements were performed using an electrical bioimpedance device (1500MDD II, Bodystat, Douglas, Isle of Man, British Isles), which required subjects to fast for approximately 4 h, have an empty bladder, and metallic objects removed.

Statistical analysis. Quantitative variables are expressed as mean and standard deviation or median and interquartile range according to their distribution; the Kolmogorov–Smirnov and Shapiro–Wilk tests were used to corroborate the normality of the distribution of the variables. Anthropometric characteristics were compared among the groups, and the relationship between anthropometric variables (i.e., weight, BMI, NC, MUAC, WC, and TSF) and %BF obtained by BIA was determined using Pearson or Spearman correlation tests depending on the distribution of each variable. Subsequently, the same analysis was performed separately for each group (i.e., normal weight, overweight, and obese). Multivariate models were constructed with %BF as the dependent variable and anthropometric parameters as independent variables. Statistical analysis was performed using SPSS version 20.0 (IBM Corporation, Armonk, NY, USA).

This study was approved by the hospital’s ethics and research committee. Informed consent was obtained from all subjects involved in the study.

## 3. Results

In total, 116 children were included in the present study. However, 4 with BMI values below—2 SDs were excluded; as such, the analysis was performed with 112 subjects (71 boys, 41 girls), with a mean (±SDs) age of 7.7 ± 1.4 years. The average age of the study subjects was different between children with normal weight and overweight (*p* = 0.03); the distribution by sex was not different between groups. The mean ages of the mothers and fathers were 32.5 and 36.4 years, respectively, with mean BMI values in the overweight category for both (Table 1). Among mothers and fathers, 68% and 70.5% were overweight or obese, respectively.

Data regarding anthropometric parameters are summarized in Table 2. All measurements were significantly higher when comparing the normal weight and overweight categories and between overweight and obesity.

Anthropometric and body composition variables were compared by sex, and a significant difference was found only in the values of neck circumference, being greater in boys (median of 28.6 vs. 26.5 cm; *p* = 0.02).

Correlations between %BF evaluated using BIA, NC, and other anthropometric variables are summarized in Table 3. In the entire group, there was a direct and significant correlation between %BF and NC (r = 0.50, *p* < 0.001). However, this relationship lost statistical significance in the case of normal weight when separated according to body weight category. The relationship maintained its significance in subjects from the overweight and obesity groups; however, the r values were higher when combining these two groups (overweight and obesity, r = 0.37), probably due to the greater number of subjects in the analysis (*n* = 62); as such, the results are reported in this way.

In the entire group (*n* = 112), %BF demonstrated a direct and significant correlation with all anthropometric parameters, being highest with BMI (0.73 for raw values and 0.71 for *Z* score). Again, these correlations were lower, and some were non-significant in subjects with normal weight; only BMI (both the raw value and the *Z* score) and TSF exhibited significant relationships, but with lower r values than for the total and the overweight/obesity groups. In the overweight/obese group, the correlations between %BF and all anthropometric parameters were direct and significant, with the highest values for BMI and WC as anthropometric indicators of adiposity. When analyzing the correlations by sex in the entire group, we again found differences with a higher r value in boys (0.67) than in girls (0.30) for the correlation between NC and %BF.

Finally, a multivariate analysis with %BF as the dependent variable and anthropometric indicators of adiposity as independent variables was performed. In all cases, BMI exhibited the highest correlation, with R^2^ values of 0.50, followed by WC and MUAC (R^2^ = 0.49 in both cases). For NC, the R^2^ value was 0.30. Adjusted for all anthropometric variables, the R^2^ value for this model was 0.57, while for the model that included only NC and MUAC, R^2^ was 0.44 (Table 4). Adjustment for sex did not modify the values reported in the model.

## 4. Discussion

Obesity continues to be among the most significant public health problems worldwide, thus justifying efforts to identify indicators of this disease and its complications [1,20,21].

For >20 years, BMI has been considered the best indicator for its identification, both clinically and in population studies, owing to its correlation with fat mass and its accessibility for use in clinical practice and in the community [22,23,24]. However, some of its limitations have led to the evaluation of other anthropometric measures to identify those with excess body fat and considered indicators of metabolic risk, such as WC, WHR, and NC.

Our results revealed that NC was a good indicator of adiposity because it correlated appropriately with %BF, although was not superior to BMI or WC, which exhibited higher correlation values in all cases, confirming them as the best anthropometric indicators of adiposity [25], even in the multivariate model in which the main contributor was BMI followed by WC.

Interestingly, NC did not exhibit a correlation with %BF in normal weight subjects. In the opinion of the authors, the age of the study subjects (5–10 years) was probably an influence in this regard given that, the accumulation of fat in the neck region should be minimal at this age and, therefore, not an accurate indicator of body fat, in addition to normal weight status in which fat accumulation is not excessive. In contrast, NC exhibited a direct and significant correlation with %BF in overweight and obese participants; therefore, it could be considered an adequate indicator of adiposity in children in these body weight categories. Similar to other studies, we found a direct correlation between NC and other anthropometric indicators of adiposity [13,17] that was stronger when evaluating the entire group than when stratifying the subjects according to body weight category.

Furthermore, as shown in the results, we tested a model with %BF as the dependent variable and NC and MUAC as independent variables because they are two measurements that do not require removing clothing for measurement and yielded an adequate correlation (R^2^ = 0.44), which was higher than that obtained from NC but lower than that of MUAC, which had values close to BMI and WC. It is important to note that parameters, such as NC and MUAC, have the advantage of not requiring more than a tape measure for evaluation, which makes them more accessible than BMI. This can facilitate community evaluations where they could be used as screening tests for the diagnosis of overweight and obesity, with the purpose of referring those who exceed an established cut-off point for a complete evaluation [7,11].

Few studies have evaluated the relationship between NC and %BF evaluated by BIA. In a cohort study from the United States, Phan et al. [26] evaluated 75 predominantly Caucasian subjects (only 7% Hispanic) with obesity (mean age 13 ± 2.4 years) for the purpose of evaluating the correlation between changes in anthropometric measurements and fat mass evaluated using BIA in a follow-up ≥3 months. The only indicator that reflected changes in fat mass was BMI.

In 2013, Bammann et al. [16] evaluated 78 children 4–10 years of age from four countries in Europe, 35.8% of whom were overweight or obese. Neck circumference had an R^2^ (unadjusted) value of 0.48 as a predictor of fat mass in a three-compartment model that included the use of BIA for the measurement of body mass. In multivariate models that included NC, WC, hip circumference, and arm circumference measurements, R^2^ increased to 0.88, with WC as the main component of the model. Andrade et al. [9] evaluated 2794 children and adolescents (6–19 years of age) in Brazil with a mean age of 11.1 ± 3.1 years and, in a manner similar to the present study, reported a direct and significant correlation between NC and BMI, and WC and %BF evaluated using BIA at all ages, with higher values in females than in males.

The differences that we observed when the analysis was carried out by sex have already been reported by other authors [9,10], although the results are not consistent with those of the present study. Andrade et al. reported higher NC values in boys, although in those over 10 years of age; furthermore, both in the report by Andrade et al., as in that of Kim et al., the correlation values between NC and %BF were higher in girls. It would be necessary to study a larger number of children to evaluate these differences.

Neck circumference is a simple, rapid, low-cost measurement that is not influenced by fasting conditions, clothing, ambient temperature, or sociocultural limitations [18]. It also has the advantage of not requiring the removal of clothing for evaluation, even partially as required to measure WC, which is especially useful in individuals stigmatized by their body weight, who may also have a phobia of weighing themselves, and in circumstances in which removing clothing to measure WC is not feasible or is uncomfortable. Furthermore, NC has been consistently associated in practically all studies, including the present work, with markers of general and central adiposity, such as BMI, WC, and waist/hip ratio, in both pediatric and adult populations [11,12,13,18,27]. However, BMI appeared to be the best indicator of adiposity in children, even during longitudinal follow-up, regardless of age, sex, ethnicity, and limitations [25,28].

The present study had some limitations, including its cross-sectional design, which precluded us from drawing causal inferences, the small number of subjects included, subjects who were identified in a hospital unit, and the fact of determining correlations with other indirect indicators of adiposity, such as BIA, which, although is a useful method and practical for estimating body fat, has limitations regarding its precision compared to other measurement methods [29,30,31]. However, in everyday practice, it is not easy to estimate body fat using more precise methods, such as dual X-ray absorptiometry or other models with ≥3 components.

## 5. Conclusions

Neck circumference exhibited a consistent relationship with other anthropometric indicators that indirectly reflected adiposity in school-age children and had an adequate correlation with %BF evaluated using BIA, particularly in overweight or obese subjects. Therefore, NC would be useful in the screening of population groups with the advantage of not requiring any specialized instruments for its measurement other than a tape measure. Its role as an indicator of metabolic risk is still being studied; however, according to our results, it could be considered in overweight or obese subjects, although it is not currently recommended for use as an isolated parameter. Body mass index and waist circumference were the best indicators of general and central adiposity, respectively.

## Figures and Tables

**Table 1:** Mean age of children and their parents, and parents’ BMI (*n* = 112).

Variable	Mean ± SDs	Median	Minimum	Maximum
Age (years)	7.7 ± 1.4	7.8	5.1	10.2
Mother’s age (years)	32.5 ± 5.9	31.0	22	55
Mother´s BMI (kg/m^2^)	28.8 ± 6.6	28.4	16	56.2
Father´s age (years)	36.4 ± 7.7	35.0	24	60
Father´s BMI (kg/m^2^)	28.8 ± 5.4	27.9	20	47

**Table 2:** Age, anthropometric, and body composition variables ^1^ according to body weight category ^2^.

Variable	Normal Weight (*n* = 50)	Overweight (*n* = 25)	Obesity (*n* = 37)
Age (years)	7.33 ± 1.3	8.2 ± 1.3	7.8 ± 1.4
Weight (kg)	22.1 (20.1–25.9)	30.4 (28.0–35.3)	40.9 (34.9–49.6)
Height (cm)	122.4 (115.4–127.2)	127.3 (123.4–133.2)	130.0 (122.2–136.4)
Height (*Z* score)	−0.54 (−1.06–0.46)	0.28 (−0.61–0.52)	0.58 (0.08–1.7)
BMI (kg/m^2^)	15.3 (14.4–16.0)	18.9 (17.8–19.6)	23.7 (21.3–26.7)
BMI (*Z* score)	−0.21 (−0.95–0.38)	1.55 (1.08–1.82)	3.1 (2.6–3.9)
Mean arm circumference (cm)	17.0 (15.8–18.4)	20.8 (19.0–22.6)	25.0 (23.2–26.9)
Neck circumference (cm)	26.2 (25.5–27.0)	29.0 (26.8–29.6)	31.0 (29.4–33)
Waist circumference (cm)	53.6 (51.2–57.2)	61.7 (57.5–69.9)	74.6 (67.8–82.2)
Tricipital skinfold thickness (mm)	9.0 (7.0–11.0)	15.0 (11.0–19.5)	23.0 (15.0–28.0)
Body water (%)	57.9 ± 6.2	52.9 ± 5.7	46.3 ± 6.2
Lean mass (%)	73.5 ± 3.9	65.4 ± 6.1	60.7 ± 8.0
Fat mass (%)	24.9 ± 7.7	31.3 ± 7.3	39.8 ± 8.1

^1^ Median and interquartile range, or mean and standard deviation; ^2^ *p* = Kruskal–Wallis or ANOVA < 0.001 for anthropometric and body composition variables; *p* = 0.03 for age (post hoc analysis normal weight vs. overweight *p* = 0.037).

**Table 3:** Correlations (Spearman) between neck circumference values and other anthropometric parameters with body fat percentage.

	Entire Group (*n* = 112) ^1^		Normal Weight (*n* = 50)		Overweight and Obesity (*n* = 62)	
	Neck Circumference	Fat Mass (%)	Neck Circumference	Fat Mass (%)	Neck Circumference	Fat Mass (%)
Fat mass (%)	0.50	–	−0.08	–	0.37 ^3^	–
Neck circumference	–	0.50	–	−0.08	–	0.37 ^3^
Body weight	0.77	0.63	0.58 ^1^	0.03	0.60 ^1^	0.51 ^1^
MUAC	0.79	0.67	0.64 ^1^	0.07	0.66 ^1^	0.62 ^1^
Waist circumference	0.74	0.69	0.55 ^1^	0.02	0.66 ^1^	0.66 ^1^
TSF	0.61	0.69	0.32 ^2^	0.35 ^3^	0.40 ^3^	0.52 ^1^
BMI (kg/m^2^)	0.76	0.73	0.62 ^1^	0.24 ^3^	0.54 ^1^	0.66 ^1^
BMI (*Z* score)	0.73	0.71	0.56 ^1^	0.30 ^3^	0.50 ^1^	0.63 ^1^

^1^ *p* < 0.001 (in all cases for the entire group); ^2^ *p* < 0.05; ^3^ *p* < 0.01.

**Table 4:** Multivariate models with body fat percentage as dependent variable.

Dependent Variable	Independent Variables	R^2^	R^2^ Model 1 ^2^	R^2^ Model 2 ^3^
Fat mass (%)	BMI (*Z* score)	0.50	0.57	0.44
	Body weight	0.37		
	Body weight category ^1^	0.41		
	Neck circumference	0.30		
	Arm circumference	0.49		
	Waist circumference	0.49		

^1^ Normal weight, overweight, and obesity. ^2^ Model 1: adjusted to all variables (*p* < 0.001). ^3^ Model 2: NC and MUAC as independent variables (*p* < 0.001).

## Data Availability

The data presented in this study are available on request from the corresponding author due to the handling of the data by the authors for other academic purposes.
